# Assisted technology in Parkinson's disease gait: what's up?

**DOI:** 10.1055/s-0043-1777782

**Published:** 2024-02-23

**Authors:** Tamine T. C. Capato, Janini Chen, Johnny de Araújo Miranda, Hsin Fen Chien

**Affiliations:** 1 Universidade de São Paulo, Faculdade de Medicina, Departamento de Neurologia, Centro de Distúrbios do Movimento, São Paulo SP, Brazil.; 2 Radboud University Medical Centre, Donders Institute for Brain, Cognition and Behavior, Department of Neurology, Nijmegen, The Netherlands.; 3 Universidade de São Paulo, Faculdade de Medicina FMUSP, Departamento de Ortopedia e Traumatologia, São Paulo, SP, Brazil.

**Keywords:** Parkinson Disease, Gait Disorders, Neurologic, Neurological Rehabilitation, Self-Help Devices, Virtual Reality, Exoskeleton Device, Physical Therapy Modalities, Doença de Parkinson, Transtornos Neurológicos da Marcha, Reabilitação Neurológica, Tecnologia Assistiva, Realidade Virtual, Dispositivo Robótico, Exoesqueleto, Fisioterapia

## Abstract

**Background** Gait disturbances are prevalent and debilitating symptoms, diminishing mobility and quality of life for Parkinson's disease (PD) individuals. While traditional treatments offer partial relief, there is a growing interest in alternative interventions to address this challenge. Recently, a remarkable surge in assisted technology (AT) development was witnessed to aid individuals with PD.

**Objective** To explore the burgeoning landscape of AT interventions tailored to alleviate PD-related gait impairments and describe current research related to such aim.

**Methods** In this review, we searched on PubMed for papers published in English (2018-2023). Additionally, the abstract of each study was read to ensure inclusion. Four researchers searched independently, including studies according to our inclusion and exclusion criteria.

**Results** We included studies that met all inclusion criteria. We identified key trends in assistive technology of gait parameters analysis in PD. These encompass wearable sensors, gait analysis, real-time feedback and cueing techniques, virtual reality, and robotics.

**Conclusion** This review provides a resource for guiding future research, informing clinical decisions, and fostering collaboration among researchers, clinicians, and policymakers. By delineating this rapidly evolving field's contours, it aims to inspire further innovation, ultimately improving the lives of PD patients through more effective and personalized interventions.

## Resumo

**Antecedentes** Os distúrbios da marcha são sintomas prevalentes e debilitantes, diminuindo muito a mobilidade e a qualidade de vida dos indivíduos com doença de Parkinson (DP). Embora os tratamentos tradicionais ofereçam alívio parcial, há um interesse crescente em intervenções alternativas para enfrentar esse desafio. Recentemente, um aumento notável no desenvolvimento de tecnologia assistida (TA) foi testemunhado para ajudar indivíduos com DP.

**Objetivo** Explorar o cenário crescente de intervenções de TA adaptadas para aliviar deficiências de marcha relacionadas à DP e descrever as pesquisas atuais para esse fim.

**Métodos** Nessa revisão, pesquisamos artigos em inglês publicados no PubMed de 2018 a 2023. Além disso, os resumos de cada trabalho foram lidos para assegurar a sua inclusão. Quatro pesquisadores buscaram independentemente os artigos de acordo com os critérios de inclusão e exclusão.

**Resultados** Incluímos trabalhos que preencheram os critérios de inclusão. Identificamos as tendências em tecnologia assistiva na análise dos parâmetros da marcha em DP. Esses compreendem os sensores portáteis, análise da marcha, retroalimentação em tempo real e técnicas de pista, realidade virtual e robótica.

**Conclusão** Essa revisão é um recurso para orientar pesquisas futuras, informar decisões clínicas e promover a colaboração entre pesquisadores, médicos e formuladores de políticas. Ao delinear os contornos deste campo em rápida evolução, pretende inspirar mais inovação, melhorando em última análise a vida dos pacientes com DP através de intervenções mais eficazes e personalizadas.

## INTRODUCTION

Parkinson's disease (PD) is a progressive neurodegenerative disorder affecting millions of individuals worldwide, significantly burdening patients and their caregivers. 1 Among the myriad of symptoms associated with PD, gait disturbances are one of the most prevalent and debilitating, contributing to a substantial decline in the quality of life (QoL). 2 Although many resources are available for the management of PD motor symptoms, gait impairment only partially benefits from antiparkinsonian drug treatment and surgical intervention. 1 3

Assistive Technology (AT) is an umbrella term covering the systems and services related to the delivery of assistive products (AP) and services, enabling, and promoting the inclusion and participation of persons with disability, aging populations, and people with non-communicable diseases. Therefore, the use of AP may maintain or improve PD patients' functioning and independence, thereby promoting their well-being (WHO assistive - https://www.who.int/health-topics/assistive-technology#tab=tab_1 ). AT may be an asset in the rehabilitation program 4 and development of devices enabling objective, accurate, and better gait assessment and monitoring is crucial for people with PD. 5

In response to this pressing need, novel rehabilitation interventions and technology have emerged, offering new avenues for assisting individuals with PD in managing their gait difficulties. 6 7 The search for innovative interventions has led to the surge of numerous gait AP such as wearable devices, 8 robotic exoskeletons, 9 10 Robot-assisted and treadmill, 11 virtual reality (VR), 12 13 exergames-based interventions, 14 smartphone or mobile health applications, 15 and sensor-based systems 16 among others. These AP, with diverse mechanisms of action, provide real-time feedback on gait parameters, promote targeted rehabilitative exercises, and may enhance overall gait performance. 5 15

A better understanding of emerging AT to assess gait in PD is important since it continues to advance and become more available. However, keeping pace with rapidly evolving technologies is challenging. Recognizing the potential and utility of these AP for assessing or improving mobility will help consumers and researchers to better manage and broaden their applicability in rehabilitation or clinical practice.

Herein, this review aims to provide an overview of current AT interventions for PD gait rehabilitation. We hope to enable healthcare professionals to understand the available technologies and their clinical and rehabilitation applications. For this purpose, we compiled and synthesized the existing body of literature and selected studies that assessed the efficacy, usability, and acceptability of current technological interventions. We also explored the outcomes of recently published studies, including improvements in gait speed, balance, stride length, and fall risk of PD patients.

## METHODS

Given the limited scientific evidence and the heterogeneity of the available studies in this field, we opted for a review to give an overview of the emerging body of literature on assistive devices to improve gait in PD.

### Identification of studies, selection, and eligibility

This review was written according to the recommendations of Preferred Reporting Items for Systematic reviews and Meta-Analyses extension for Scoping Reviews (PRISMA-ScR). 17

We searched on PubMed studies characterized as clinical trials (randomized and non-randomized), meta-analyses, systematic reviews, literature reviews, cross-sectional studies, cohort studies, and pilot studies. We looked for the following descriptors: assistive technology, assistive device, Parkinson's disease, gait disorders, gait analysis, wearable sensors, wearable devices, cueing, virtual reality, exergames, robotic-assisted gait.

**Figure 1:**
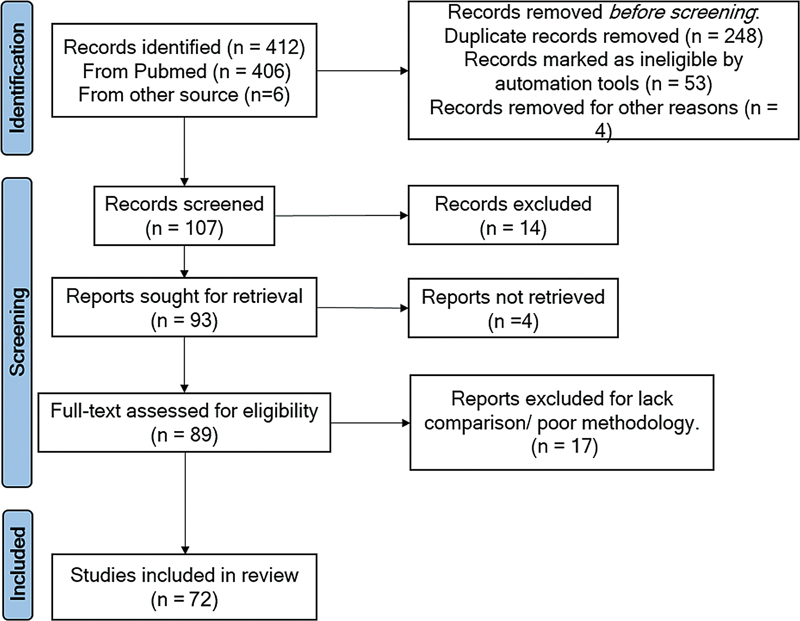
Flow diagram of literature search strategy.

We included only studies published in English and human subjects between October/2018 and October/2023. Additionally, the abstract of each study was read to ensure inclusion. All studies that did not mention Parkinson's disease or present technologies to assess or treat PD in the methodology were excluded ( Figure 1 ).

Four reviewers (T.C., J.C., J.A., H.C.) screened titles and abstracts by inclusion and exclusion criteria. The authors worked in pairs and solved disagreements (if there were any) before the study inclusion.

## RESULTS

We only included studies that met all inclusion criteria after analyzing their respective titles, abstracts, and texts. In total, we selected 412 studies. We included 72 studies that met all inclusion criteria. Several types of technological devices used to evaluate and treat gait in Parkinson's disease were identified.

We found several assistive technology devices for gait analysis, and they are used to identify and diagnose gait and other motor symptoms (EEG, EMG, Azure Kinect, HTC VIVE VR; GAITRite Portable Walkway System), 18 19 20 21 to track gait and motor symptoms (VIBE), 22 23 and to treat gait (Keeogo Reha exoskeleton, Tymo system; Treadmills, Gait Trainer GT1, Lokomat, Cycle Ergometers, Walkbot-S, Non-motorized treadmill, G-EO system, SMART Lounge vibroacoustic system, Smart shoes, XaviX system, App “PatientConcept”). 18 24 25 26 27 28 29

The most frequently used systems in gait analysis are motion capture systems (3D systems, multi-sensors combined or optoelectronic systems, 3D systems, multi-sensors combined or optoelectronic systems), 20 21 inertial Measurement Unit (IMU) 20 30 ; and force plates. 20 Other studies monitoring gait parameters in PD used wearable sensors or wearable devices based on different types of sensors. 31 32 33 34 35 36 37 38 39

Two guideline reviews emphasized the high level of evidence of cueing techniques using real feedback to improve gait performance. 3 6 We also found some studies using devices as compensation strategies to improve gait in PD such as visual laser cues, 40 rhythmical auditory cues provided by a metronome, 41 and tactile cues. 42

Some studies used VR to interact three-dimensional virtual environment. 4 25 43 44 45 46 47 48 49 50 51 52 53 54 55 56 57 58

We also found studies using Robotic-assisted gait training for PD. 24 29 59 60 61 62

We analyzed several types of AP and categorized them into commercial and non-commercial devices available for research, rehabilitation, or home-based care. The majority of them are available on the market and depicted in Table 1 . To make for easy reading, and based on the interventions that were described, we subdivided our findings into the following subtopics: wearable sensors, gait analysis, real-time feedback and cueing techniques, VR, and robotics.

**Table 1:** Overview of tradeable and non-tradeable devices

Type of assistive products	Devices	Manufacturers of commercial and non-commercial devices available
Apps	PatientConcept	NeuroSys GmbH
	ListenMee APP	Human Bionics SAS
	Net MD system	Smartwatch3 SWR50 model, Sony
Gait and balance systems	3-D Motion Capture systems	ActiGraph LEAP™
	EquiTest Computerized Dynamic Posturography unit	NeuroCom International Inc.
	Force Plate	NI
	Force Sensitive Resistor (FSR)	NI
	GAITRite Portable Walkway System	CIR Systems Inc.
	Inertial Measurement Unit (IMU)	Heel2Toe TM Shimmer Research Ltd.
	Multi-sensors combined	CuPiD System Project
	Optoelectronic systems	NI
	Smart-EquiTest Balance Master system	NeuroCom International Inc.
	Tymo system	Tyromotion Inc.
	VICON motion capture system	Vicon Motion SystemsLtd
	Mobile Motion Visualizer	System Friend
Exoskeletons	Walkbot-S	P&S Mechanics
	Keeogo Rehab exoskeleton	B-Temia Inc
	Stride Management Assist exoskeleton	Honda R&D Co., Ltd
	Yorisoi robot	Sanyo Homes
Robot-assisted walking	G-EO system	Reha Technology AG
	Gait Trainer GT1	RehaStim Medtec AG
	Lokomat	Hocoma
Virtual reality equipments	Microsoft Kinect and Xbox gaming console	Microsoft Inc.
	HTC VIVE VR	HTC and Valve Corporation
	Nitendo wii	Nintendo Co., Ltd.
	Tymo	Tyromotion Inc.
	MOTIGRAVITY system	NI
	V-TIME Project equipamento	NI
	Smart shoes	JiBuEn gait analysis
Insole sensors	Insole	BalancePRO
	Smart insole + app	Walk With Path Ltd.
	Moticon sensor insole	Moticon ReGo AG
Others	Electroencephalography (EEG)	NI
	Electromyography (EMG)	NI
	Google Glass	Google Inc.
	Non-motorized treadmill	NI
	Recumbent Cycle Ergometers	NI
	SMART Lounge vibroacoustic system	NI
	Smart shoes	NI
	Smartphone	NI
	Treadmills	NI

Abbreviation: NI, not informed.

## DISCUSSION

### Wearable sensors

The advancement of sensor technology, motion capture systems, and force platforms have been employed as more objective and precise methods for evaluating PD patient's gait. 33 Sensor and Wearable devices (SWD) facilitate and monitor an individual's activity. They are an important asset for intervention and monitoring and are easy to integrate into the patients' daily routines. 31 SWD enables the remote assessment of patients' conditions in their natural environment, promoting a more comprehensive clinical evaluation and empowering patients to monitor their gait.

Most of the SWD sensors to analyze spatio-temporal parameters of gait in patients with PD were placed on both ankles and tibias, feet, chest, and lower back. For gait parameters, speed was the most common feature evaluated, followed by cadence, stride length, and stride time. For step characteristics, step time, step length, and right-left asymmetry were mostly assessed. 32

Many low-cost devices have been employed to collect real-time spatiotemporal, kinematic, and kinetic gait features in PD patients. Costly force platforms used to measure ground reaction force during gait may be replaced by pressure insole. These insoles are composed of an accelerometer, gyroscope, and sometimes a magnetometer attached to the patient and can measure the linear and angular velocity, acceleration, and other gait parameters. 35 Sensors may also be placed under smart shoes and inertial microelectromechanical system sensors attached to the patients to measure stride length, gait velocity, range of motion of the ankle, knee, and hip joints of individuals with PD. 36 Other studies are focusing on developing new SWD to detect the occurrence of freezing of gait (FOG) and its discrimination in PD (freezing, shuffling, and trembling), 37 and promising resources may be launched in the near future. 38

These AP have also been tested to discriminate PD individuals from healthy controls. A recent study explored which parameter could discriminate PD from controls, and they observed that kinematic features were more informative than kinetic analysis. 39 Smartphones are a promising tool to discriminate gait and postural instability between healthy controls and individuals with PD. 63 However, it has also been used to promote home-based gait training, so future research should investigate the role of smartphones as an AP for home-based rehabilitation support.

Moving arms in a rhythmic and symmetrical contributes to postural stability and walking efficiency. SWD may also be placed on the upper limb and are useful to measure the arms arm swing during walking. One trial used a motion-capture system computer with speech models and advanced artificial intelligence sensors for orientation and spatial tracking to collect 3-dimension joint trajectories and body segments during walking. The authors could quantify arm swing features as well as lower gait velocity of PD patients compared to healthy controls, thus offering a simple tool to provide a more comprehensive gait assessment. 64 65

A recent consumer-centered review described the available commercial SWD targeting improvement in gait patterns and walking behavior. Of the 11 commercially available devices, only 4 (36%) had findable evidence for efficacy potential supporting the claims. 34 Although there are promising results with SWD for gait assessment, many challenges need to be overcome. To cite some, gait requires complex algorithms for its analysis, and most of the available portable devices do not avoid noise contamination or provide precise data synchronization and collection of real-time spatiotemporal, kinematic, and kinetic parameters. Available SWD capture short walking bouts and good gait quality measures cannot rely on short interval data. Moreover, gait assessment obtained from laboratory machine learning methods does not necessarily reflect gait features in patients' daily living. New algorithms and sensor detectors resolution improvements are needed to accurately extract facets of gait quality and function. 64 Future devices should be sensitive to precisely distinguish between healthy controls and early or mild symptomatic PD patients and also provide accurate gait parameters enabling early intervention of patients with a higher risk for falling or FOG.

### Gait analysis

Emerging technology for gait analysis aims to identify and diagnose abnormal gait parameters in PD, but it may also be used to monitor and improve gait, balance, and mobility. 7 Currently, most of the devices used for gait analysis can be categorized into a) motion capture systems (3D systems, multi-sensors combined or optoelectronic systems); b) inertial measurement unit; and c) force plates. 20 22 30 66

Most of the trials for gait analysis evaluated its speed with good reliability. The Berg Balance Scale and UPDRS part III were often used for outcome ratings. Overall, the AP were well accepted by the patients. 10 18 22 23 24 25 26 27 Other parameters frequently assessed in the studies besides gait speed were: step length, step symmetry, falls and freezing of gait. 28 29 30 66 Assistive devices obtained significant correlations on PD gait analysis in at least one of the above-mentioned parameters. However, the effectiveness of these AP and the outcomes are variables since they cannot yet overcome laboratory gait conventional monitoring or replace validated PD gait assessment tests or scales. Still, some commercial devices have been efficacious for the analysis and treatment of gait and balance in PD. 10 18 20 27

Many AP devices not only assess but are also ancillary for gait and balance rehabilitation treatment. In this aspect, gait assistance robots (GAR), such as the G-EO system, Gait Trainer GT1, Lokomat, and GAITRite are the preferred equipment for this purpose. 10 18 27 29 Another gait treatment strategy is to combine traditional treadmill training with assessment assistive devices. 18 Before long, more walking aid gadgets and innovative AP will be launched on the market combining both evaluation and treatment strategies 28 and more trials will be necessary to explore their plausibility and effectiveness in clinical practice.

Despite the promising potential of assisted technology for gait analysis, several challenges and questions remain unaddressed. 67 Several barriers may be listed for its limited utilization as a gait rehabilitation tool such as high cost, unfamiliarity with technology, technical issues, low usability, and so on. Being so, strategies to increase awareness and make this resource generally available are welcome and may help to broaden assistive devices accessibility for research, clinical, and consumers use. As an example, in the last years, there has been an increasing process for portability and miniaturization of mobile health technologies. 7

Furthermore, ethical concerns regarding data privacy, patient autonomy, and equitable access to technology must be examined in-depth. Additionally, the use of these devices and outcomes on the role of telehealth and remote monitoring in delivering personalized gait analysis programs requires further exploration, especially in the context of evolving healthcare practices and digital health solutions. So, due to the complexity of PD gait management, perhaps advances in artificial intelligence may, in near future, promote, refine analyses, and support treatment strategies in PD gait rehabilitation.

### Real-time feedback and cueing techniques (auditory, visual, and proprioceptive)

The positive effects of the cues on gait performance (speed, cadence, step length, and stride) are well recognized and supported by a robust level of evidence. 3 6 External cues, such as auditory and visual ones, are used to bypass and compensate for the brain's abnormal internal cueing mechanisms that cause deficiencies in movement planning and execution in PD. 2

These compensation strategies (or cueing techniques) delivered by physiotherapists, such as multimodal training, may provide feedback, repetition, and high challenges and can be used to correct temporal aspects of gait, improve balance and walking speed and prevent freezing episodes. 41 It has been demonstrated that people with PD training with audio cues, such as a digital metronome improve balance and gait performance. 41 Besides, cueing training may overcome or ameliorate FOG episodes in the short and long term. 68 Moreover, cueing may be applied to different mobile health applications, 15 and digital devices such as robot-assisted rhythmic cues 59 or laser attached to patient's shoes (the laser shoes). 40

Additionally, proprioceptive devices, such as tactile cues or vibratory ones, are being explored to provide external support during walking. 42 Finally, visual, and auditory cues can be combined with treadmill training, and it has shown to be better than standard treadmill training alone. 46 This association may focus on gait and cognitive deficits to optimally address several critical aspects of fall risk and improve mobility, physical activity, cognitive function, and FOG in PD.

### Virtual reality

VR is a computer technology that enables users to interact in a three-dimensional (3D) virtual environment and experience similar situations in the real world. This technology facilitates the perception of physical movements and visual, auditory, and tactile input. VR can be categorized into three types according to the degree of immersion: non-immersive, 4 semi-immersive, 52 and full-immersive. 63 In a non-immersive system, the patient can interact with the virtual environment using a computer screen and game console. 4 A semi-immersive VR system consists of large screens or projections to enable a visual-virtual 3D-space experience and uses interactive devices such as a motion tracker, haptic gloves, and balance platform. 52 Fully immersive VR incorporates the combination of more sophisticated graphic systems to create a virtual world and advanced wearable devices. It allows users to safely and effectively experience motor challenging situations at their own pace and difficulty level and can observe their performance in real-time, receiving immediate feedback regarding movement and balance control. 69

Exergame is the gamification of rehabilitation using electronic games that capture and simulate real movements. It requires the participants to be physically active or exercise and apply full body motion to play the games. 70 It is a low-cost and accessible approach for healthcare professionals to incorporate this training in rehabilitation programs. Examples of exergames resources are Nintendo Wii, Kinect, and games designed for computers or sensors like Leap Motion. 70 Therapy based on commercial video games increases patients' motivation, provides direct feedback, and allows dual-task training. 49 This exercise modality appears to benefit functional circuitry in PD and facilitates learning through reinforcement feedback. 69 The training approach promotes sensory input, central integration, coordination, weight transfer, and balance, with subsequent improvements in muscle control and coordination, and might improve motor function in neurological disorders. 53

Studies have shown that short-term exergame associated or not with conventional training may improve QoL, 43 postural instability, balance, 44 54 mobility, gait parameters, and reduced risk of falls in PD. 25 These findings were endorsed by a systematic review in which exergame training reduced the number of falls and improved static and dynamic balance in patients with PD. 4 It is worth highlighting that in order to improve balance, a supervised VR program might require at least 20 minutes, 4 to 6 times a week of training 54 and that younger patients have better outcomes than older ones. 55 The longer-term efficacy of VR and exergames interventions is still unclear, therefore, more trials are needed to address these questions.

Immersive VR with body weight supported treadmill warrants PD patients to exercise in a safer condition. It provides repetitive, task-oriented, and higher-intensity training. The association of both apparatuses with a 20% body weight support improves gait and balance assessment. 58 Moreover, those who trained with the treadmill and VR had fewer falls than those with the treadmill only, but no changes were found for FOG. 45 The benefit may vary according to training duration, PD patients who underwent a 12-week exercise program had better gait speed, stride length under dual-task conditions, and falls reduction compared to a 6-week VR training. 46

So far, most of the studies evidenced that VR rehabilitation may have beneficial effects on balance. In relation to gait, improvements in speed, step length, and cadence in both kinematics and clinical scale tests were observed, 49 but no significant differences were seen in walking speed in PD. 56 On the other hand, a meta-analysis showed no statistically significant differences in gait ability, activity of daily living, motor function, and QoL in PD after VR training. 57 It is noteworthy that a meta-regression analysis utilizing publication year as the predictor variable showed a greater improvement in balance function in recent studies compared with older ones. This could be attributed to the technological advancement. VR has become cheaper and more accessible, enabling more patient enrollment in trials and resulting in greater data accessibility.

Studies observed that VR rehabilitation may improve neuroplasticity and change brain neural patterns by activation and reorganization mainly of the primary motor cortex, sensorimotor cortex, and supplementary motor area. 48 In addition, functional magnetic resonance imaging showed that PD patients who underwent VR training increased activity in the precuneus region, which is linked to visuospatial integration, memory, and self-awareness, and this network is active during activities involving memory encoding and retrieval. 47 Cognition plays an important role in postural control and may interfere with gait and posture assessment and treatment. 71 So, advances in artificial intelligence (AI) may promote and refine strategies for VR in PD rehabilitation. It will allow more precise interventions since gait is very complex and an excessive number of features requires robust computing power to obtain more accurate gait performance analysis. 72 This analysis could help detect the onset of walking abnormalities, revealing the transition from normal to pathological gait.

Besides AI will offer better resolutions of patients' data, and identify parameter changes and PD symptoms and severity, which may be shared across healthcare networks and members of the interdisciplinary team, offering more personalized treatment. 15 PD rehabilitation could benefit in near future from a more effective approach with more challenged and realistic virtual environment. 52 Finally, VR rehabilitation should be offered to PD patients as a supplementary approach to existing proven and safe interventions until further and more robust evidence is available. 50 51 53 57 Hence, large trials with good methodologic designs are necessary with a focus on comparing the efficacy of VR-based rehabilitation with conventional treatments and or new emerging technologies.

### Robotics

Robotic-assisted gait training (RAGT) has been employed for PD patients as a complementary approach to conventional rehabilitation treatment. 61 These AP devices support the body weight, control body sway and improve the safety profile, which may benefit balance, postural control, and gait parameters. 10 Repetitive locomotor training may also facilitate neuromuscular regulation, provide proprioceptive cueing effect, shift the body weight from one leg to the other, reduce muscle co-contraction, improve contraction/inhibition patterns, and strengthen lower limbs. The gait-like movement may also have a positive effect on the gait central pattern generators at the spinal level. 27 29

There are a variety of robots utilized for rehabilitation including exoskeletal robots equipped with treadmills and end-effector robots. Robotic treadmills, such as the Lokomat gait training system, improve gait performance, 27 and can also be associated with VR to increase cognitive flexibility and attention shifting, as well as in executive and visuospatial skills. Visual cueing, displaying incentive messages on a screen, and auditor feedback when training with RAGT, are resources to improve the rhythm of the gait of individuals with PD. 59 An automated wearable exoskeleton robot may detect the hip joint angle and provide torque to assist in hip flexion and extension facilitating gait training. 60

Some studies have shown the beneficial effects of RAGT for FOG in PD. One study compared treadmill gait training with RAGT. PD patients had improvement in all the outcome measures (6-minute walk test, TUG, FOG, and QoL), but freezers experienced a better reduction in FOG with RAGT than control group training with treadmill alone. 29 The hypothesis mechanism suggests that repetitions of rhythmic limb movements could act as an external proprioceptive cue, by reinforcing the neuronal circuits that contribute to the lower limb movements. Another possibility is that proprioceptive cues might be the same as visual and auditory cues, so they might be involved in improving gait patterns. 61 Fall may also be prevented by body weightlifting wearable snuggling nursing robot. It has been shown that such a device may aid PD patients' gait performance during TUG test and offer a good level of security in preventing falls. 62

There is growing evidence suggesting that the utilization of these computerized technologies could potentially transform conventional therapies by facilitating safety and real-time assessments of PD patients. 73 This finding was confirmed by a meta-analysis study, however, RAGT does not impact on the gait velocity or walking distance of patients with PD. 74 RAGT may also benefit PD patients' gait endurance. 24 Therefore, assistant robotic device training can improve gait parameters with reduced motor workload in PD patients, 34 but more studies should be done to confirm these findings.

### Summary of evidence

This review aimed to identify and synthesize key trends in AT. New technologies may be used for assistance, diagnosis, monitoring, prediction of treatment response, and assistance with therapy or rehabilitation in PD patients' gait. Although many AP resources are available, only few are actually implemented in daily clinical practice. We found some hindrances to the limited use of AT and awareness of these factors may lessen the barriers between the fast-developing technological devices and consumers, making them more accessible and also broadening their implementation in PD patients' daily living or treatment.

Besides technological issues, a lack of motivation to use WSD monitoring systems is an aspect someone also must be observant of. Patients' empowerment, gaining knowledge about new AT devices, and their participation as active players in the development of research activities may also favorably increase their adherence. In addition, usability and familiarity with new devices contribute to high satisfaction of using technology in people with PD. Tailoring the best device for each individual, greater patients' gait characterization, more patient engagement and self-assessment are details to be always reminded of when selecting AP for patients' rehabilitation.

Finally, research is needed to explore the real role of AP in PD. We also need to better ascertain which technology resource will be more appropriate for gait assessment in PD. We hope his review may inspire further research, foster innovation, and to help readers to prescribe more effective and personalized technological interventions for individuals living with PD.

### Key points

- Although AT is a valuable tool for PD management, it is not widely implemented in daily clinical practice.
- Understanding of emerging technology is important and facilitates the recognition of the potential and utility of new AP for assessing and improving gait in PD.
- Patient empowerment and their inclusion as active players in the development of research activities may favorably impact compliance.
- Enjoyable, cheaper, safer, easier to use, and friendly devices may contribute to adherence to using technology.

### Limitations

Limitations for this review include missed original literature on technology and gait assessment, despite our best efforts to search for relevant articles and sources derived from our references. We hoped to provide a scope description of all original research on AT and gait however, we did not conduct a formal quality assessment of included studies and our findings could be influenced by publication bias.

In conclusion, this review provided a comprehensive synthesis of the current evidence and limitations of assisted technology in managing PD gait impairments. Assisted Technology in PD gait aspires to be a valuable resource for advancing our understanding of how technology can improve the lives of PD patients since this is a rapidly evolving field with an increasing number of publications in recent years. With the understanding of the potential of such devices and the existing research landscape, we hope to guide future investigations and inspire further research, innovation, and the development of more effective and personalized interventions for individuals living with PD.
